# Uterus: A Unique Stem Cell Reservoir Able to Support Cardiac Repair via Crosstalk among Uterus, Heart, and Bone Marrow

**DOI:** 10.3390/cells11142182

**Published:** 2022-07-13

**Authors:** Ana Ludke, Kota Hatta, Alina Yao, Ren-Ke Li

**Affiliations:** 1 Division of Cardiovascular Surgery, Toronto General Hospital Research Institute, University Health Network, Toronto, ON M5G 1L7, Canada; ana@ludke.ca (A.L.); kotahatta@gmail.com (K.H.); alina.yao@mail.utoronto.ca (A.Y.); 2 Division of Cardiac Surgery, Department of Surgery, University of Toronto, Toronto, ON M5T 1P5, Canada

**Keywords:** uterine stem cells, endometrial stem cells, stem cell reservoir, utero-cardiac axis, bone marrow-utero-cardiac cross talk

## Abstract

Clinical evidence suggests that the prevalence of cardiac disease is lower in premenopausal women compared to postmenopausal women and men. Although multiple factors contribute to this difference, uterine stem cells may be a major factor, as a high abundance of these cells are present in the uterus. Uterine-derived stem cells have been reported in several studies as being able to contribute to cardiac neovascularization after injury. However, our studies uniquely show the presence of an “utero-cardiac axis”, in which uterine stem cells are able to home to cardiac tissue to promote tissue repair. Additionally, we raise the possibility of a triangular relationship among the bone marrow, uterus, and heart. In this review, we discuss the exchange of stem cells across different organs, focusing on the relationship that exists between the heart, uterus, and bone marrow. We present increasing evidence for the existence of an utero-cardiac axis, in which the uterus serves as a reservoir for cardiac reparative stem cells, similar to the bone marrow. These cells, in turn, are able to migrate to the heart in response to injury to promote healing.

## 1. Introduction

It has long been noted that premenopausal women have a significantly lower rate of developing cardiac incidents, compared to males. However, this is not the case for post-menopausal women, where such rates are the same as that of males [1,2]. The disappearance of this disparity upon the onset of menopause has thus been the subject of significant clinical research interest [1,2]. Initially, this phenomenon was attributed to the lowered production of estrogen and progesterone after menopause; however, several studies using hormone replacement therapy (HRT) did not demonstrate any reductions in cardiac disease risk among post-menopausal women on such therapies [3,4,5]. As a result, other processes, such as cross talk between the uterus and the heart, have been investigated. Indeed, this utero-cardiac cross talk was demonstrated in our previous pre-clinical study, where uteri were implanted into adult rats, after myocardial ischemic injury [6], suggesting that a functional uterus may be an independent factor for reducing cardiac disease risk.

Owing to the fact that the menstrual cycle entails the growth, shedding, and regeneration of the endometrium in a monthly timespan in women, it has long been speculated that the addition of endometrial tissue was driven by the activities of stem cells within the uterus. This idea was first proposed by Prianishnikov, who described that an endothelial stem cell population would be an immature hormone-independent population residing in the deepest basalis layer, and with the capability of differentiation into different types of hormone-responsive endometrial cells [7]. Indeed, the presence of such cells has been verified in various mammals, particularly in mice and humans [8,9,10]. As the specific markers associated with them were mainly unknown at the time, label-retention assay, consisting of a DNA analog, bromodeoxyuridine (BrdU), being delivered, followed by a chase period, was used in mice. This results in all proliferating cells being initially labelled with BrdU, but only the non-dividing cells, dubbed the “label-retaining cells” (LRC), will maintain BrdU, likely representing the quiescent nature of stem cells [11,12,13,14,15,16]. Using this assay, as well as the histone 2B-GFP labeling assay [17,18], at least 2 major populations of LRCs, likely representing uterine stem cells, were found in mice. One of those populations consisted of stromal LRCs, found within the endometrium, and characterized by their expression of characteristic stem cell markers c-Kit and OCT-4 [11]. The mouse endometrium also contained epithelial LRCs, which were present for a shorter duration in post-natal and pre-pubertal mice, and were characterized by the lack of CD45, Sca-1, or estrogen receptor (ER) markers [14]. It is worth noting that the stromal LRCs in mice are able to undergo a “mesenchymal-to-epithelial” transition, likely serving as the basis for endometrial growth, during the estrous phase in mice [18,19]. There is also a “side population” of murine endometrial stem cells present, characterized by their ability to generate an efflux of fluorescent vital dye, such as Hoechst 33342, owing to them having ATP-binding cassette (ABC) transporters. These cells do not express any endothelial, hematopoietic, or mesenchymal stem cell markers, aside from some cells being positive for SCA-1, c-Kit, and ER-α, which indicates that these cells could differentiate into different types of endometrial cells upon estrogen stimulation [8,20].

In humans, the cell sorting techniques of FACS and MACS are used to identify uterine stem cells, which are then classified into 3 different types: endometrial stromal stem cells, endometrial epithelial progenitor cells, and endothelial stem cells [8,10,21]. Endometrial stromal stem cells are characterized by having CD44, a marker highly associated with stem cells in mice and humans, as well as CD73, CD90, CD105, CD106, and PDGFR-β [22]. Additionally, they have been found to express typical mesenchymal stem cell-associated markers, such as STRO-1 [23]. Endometrial stromal stem cells have also been found to share similar characteristics as pericytes. Pericytes are found surrounding capillaries and micro-vessels, and possess contractile properties, enabling them to play key roles in angiogenesis and regulating blood pressure [24]. Both cell types share similar adherence and morphology, as well as being localized perivascularly. Additionally, they both possess similar cell markers, such as CD146, PDGFR, CD29, CD44, CD105, CD73, and CD90 [25,26]. Furthermore, like with uterine stromal stem cells, CD146^+^ PDGFRβ^+^ pericytes can be induced to differentiate into multiple mesodermal lineages, such as adipogenic, osteogenic, and neuronal lineages. In particular, these pericytes are closely related to stromal fibroblasts, and are able to differentiate into them in vivo, suggesting that these cells belong to the same lineage [25]. Indeed, CD146^+^ pericytes, localized around capillaries and micro-vessels, within both endometrial functionalis and basalis layers [27] can re-constitute endometrial stromal tissues in vivo [28]. Therefore, pericytes are considered as the origin of endometrial stromal stem cells [26].

Endometrial epithelial progenitor cells have the epithelial cell marker CD9, SSEA-1, and N-cadherin markers, as well as long telomeres [29,30,31,32]. These cells also are able to form spheroid endometrial gland-like structures in vitro [31,32]. In fact, endometrial epithelial progenitor cells have been noted for being able to regenerate the entire glandular lineage, which is in part owed to their possessing naturally-occurring somatic mitochondrial DNA mutations at the *CCO* gene [33,34]. Another key gene is *Axin-2*, noted to be a marker of long-lived bi-potent epithelial progenitors found within endometrial glands [34,35]; cells positive for Axin-2 are able to form fully functional endometrial organoids in vitro [34,36]. As for endothelial stem cells, they possess the classical endothelial markers of CD31 and CD34 [37,38]. Furthermore, as in mice, a “side population” of ABC transporter-positive endometrial stem cells are present. These cells express CD31, CD34, epithelial membrane antigen, CD105, and CD146, as well as BCRP1/ABCG2, sharing similarities to stromal and epithelial stem cells [29]. They have also been found to be able to differentiate into multiple endometrial cell lineages, as well as being able to proliferate to a greater extent than non-side population cells [38,39], and re-form endometrium in vivo [29,30,37,40]. As a result, the side population has been regarded as a heterogeneous population harboring endometrial stem cells, as well as serving as a niche for those cells [38,40].

On top of the endogenous uterine stem cells, exogenous stem cells, originating from the bone marrow (BM), have been found in the endometrium [41]. These BM-derived stem cells (BMDSC) are a type of mesenchymal stem cell that is able to traffic out of the BM into different types of non-hematopoietic tissues [41,42]. In the case of the endometrium, BMDSCs are able to differentiate into the same type of endometrial cells, such as stromal, epithelial, and endothelial, in both mouse and human models [41,43,44]. Owing to them being mesenchymal stem cells, they express the markers CD29, CD44, CD73, CD90 and CD105, while CD14, CD34, CD45, and human leukocyte antigen–DR isotype (HLA-DR) are absent [45]. All of these populations have been found to contribute to the formation of the endometrium, indicating that these uterine stem cells, both endogenous to the organ, and those deriving from BM stem cells, could serve as a significant stem cell population for developing cell therapies.

Even though several studies have proved the existence of endometrial stem cells, the presence of cross talk with other organs has not been fully defined. In this review, we will focus on the triangular relationship among the uterus, heart, and BM, with emphasis on utero-cardiac cross talk. We also highlight the effects of this utero-cardiac axis on the development of uterine cell therapies.

## 2. The Female Advantage in Myocardial Infarction

Epidemiological studies have consistently shown that females have an advantage when it comes to myocardial infarction, in which women have lower incidence and mortality from this phenomenon compared to men [46,47,48]. Furthermore, women respond more favorably to myocardial infarction, with respect to post-infarct remodeling (healing after injury), resulting in lowered frequency and severity of heart failure compared to men, though these advantages are lost after menopause [1,2,49]. This phenomenon is of interest, as elucidation of its underlying mechanisms could possibly lead to therapeutic strategies for either preserving this advantage in post-menopausal females, or for novel treatments for heart disease in males. It has long been presumed that this loss of cardiac protection after menopause is owed to ovarian hormonal effects. However, this has been contradicted by some studies suggesting that this effect could potentially be independent of hormonal effects [3,4,5]. Therefore, a thorough examination of the putative hormonal effect is necessary, in order to rule out any possibility of it contributing to sex-based cardiovascular protection in pre-menopausal women. Firstly, in a 1956 study, Katz et al. presented compelling evidence that estrogen was cardio-protective [50], resulting in clinical trials being designed to treat men at high risk of developing cardiovascular disease with estrogen [51]. However, these male patients who were treated with a high dose of estrogen did not achieve the expected cardio-protection, and often developed the side effects of hormone therapy, such as gynecomastia, impotence, testicular atrophy, and vascular spiders [52]. The trial was discontinued as a result. Subsequently, long-term cross-sex hormone therapy trials of men receiving ovarian hormones have been conducted [53], yielding inconclusive results; the failure of the first trials was thus argued to be owed to the dose used.

In the following years, a variety of observational studies in post-menopausal women taking HRT showed some degree of cardio-protection [54,55,56]. HRT is most commonly given to post-menopausal women, in which it artificially restores ovarian hormonal levels through supplementation. Preliminary evidence suggested that the absence of hormonal stimulation served as the basis behind the cardiovascular incidences in post-menopausal women. However, these findings have been controversial, in part because many studies did not randomize HRT recipients [57]. In fact, in a randomized clinical trial, HRT actually increased the risk of coronary heart disease in the early years of its administration [58], which was further supported by additional studies [4,59]. Indeed, that randomized clinical trial was prematurely halted, as the negative outcomes of HRT outweighed the potential benefits. Therefore, many clinical studies suggested that HRT is not cardio-protective, and in fact, increases the risk of developing cardiac incidents.

Based on the HRT findings, the cardiovascular protection prior to menopause could possibly be the result of (a) ovarian function, but additionally could be dependent on (b) the presence of the uterus. In fact, the Framingham Study of 1978 by Gordon et al. was the first to report that cardiovascular protection against coronary heart disease, in terms of incidence, in women was lost when hysterectomy was performed, regardless of whether the ovaries are also removed or not [46]. These data suggest that the uterus was an independent variable affecting the risks and outcomes of myocardial infarction. This was an unexpected finding because removing the uterus, but not the ovaries, leaves ovarian hormonal function intact. A 1992 study by Palmer et al. reported similar findings [49] and cited results from other researchers demonstrating that, in some cases, hysterectomy at a pre-menopausal age could lead to ovarian failure [60]. Palmer et al. thus suggested the “hormonal hypothesis”, in which hysterectomy disrupted the proper ovarian functioning and hormonal physiology that protected the female heart [49]. However, this hypothesis, although easy to logically construct, remains difficult to prove experimentally, leading to continued controversy over its validity [61].

Gynaecologists have also noted the presence of a possible relationship between the presence/absence of the uterus and coronary artery disease [62]. However, even though a previous study has dismissed this association, and attributed the increase in adverse cardiovascular outcomes following hysterectomy to eventual ovarian failure [60], it must be noted that this refutation was part of the study’s discussion commentary, and not a conclusion reached by experimental evidence [49]. Nevertheless, the postulation that the cardio-protective contribution of the uterus is independent of ovarian function has been investigated. Falkeborn, who previously studied the effect of HRT in 1992 [56], revisited this theory in 2000 by studying myocardial infarction risks and outcomes in women who previously underwent oophorectomy (removal of ovaries) and/or hysterectomy [63]. The additional cohort of oophorectomy patients provided a previously unavailable comparison group to determine the independent contributions of ovarian and uterine physiology on pre-menopausal cardio-protection. This epidemiological study found that hysterectomy performed after natural menopause was associated with an increased risk of heart failure, suggesting that the mere presence of the uterus was cardio-protective, independently of ovarian hormones or menstruation. This finding that cardio-protection could be conferred merely by the presence of uterine tissue, even in naturally menopausal women, could serve as a possible underlying basis behind women demonstrating more favourable clinical outcomes post-myocardial infarction versus men, though further investigation is required.

## 3. Stem Cell Crosstalk between Organs

The human body is an integrated system composed of different parts that inter-communicate with each other. Various circulating molecules have been described, including hormones, proteins, cytokines, micro-RNAs, and others that either target adjacent cells or remote organs in order to preserve whole-body homeostasis [64,65,66]. Crosstalk between organs has been shown by demonstrating the trafficking of numerous types of stem cells between different parts of the body, including hematopoietic stem cells (HSC), mesenchymal stem cells, endothelial progenitors, and very small embryonic-like stem cells which circulate in the blood and maintain the pool of stem cells for regenerative purposes [67]. While the origin of these stem cell types is not well established, the trafficking of stem cells derived from the BM has been better characterized [67]. Based on our research findings, we propose that the uterus is a reservoir and exporter of reparative stem cells, in a similar fashion to that of the BM.

Ever since uterine stem cells was first described in 1978 as a hypothesis to explain the cell population kinetics of the endometrium [68], multiple stem cell populations have been identified, characterized, and examined, as outlined above. Our group has shown that uterine stem cells are able to support hematopoiesis, and that some of these stem cells are of extra-marrow origin [69,70]. Additionally, we demonstrated that uterine stem cells are recruited to the heart in response to injury and differentiate into blood vessels [6]. We propose that these phenomena can be explained by the stem cell crosstalk between organs, particularly with the utero-cardiac axis, which serves as a mechanism of reparative stem cell support for promoting cardiac healing. This concept fits well with our observation of increased mortality rates after myocardial infarction in female rats that underwent hysterectomies to levels comparable to that of males. This effect was, however, rescued by intravenous injection of uterine cells, resulting in mortality rates decreasing to that of non-hysterectomy levels, along with the restoration of cardiac function [6].

## 4. Utero-Cardiac Axis Involves a 3-Way Relationship between Heart, Uterus and Bone Marrow

**Figure 1:**
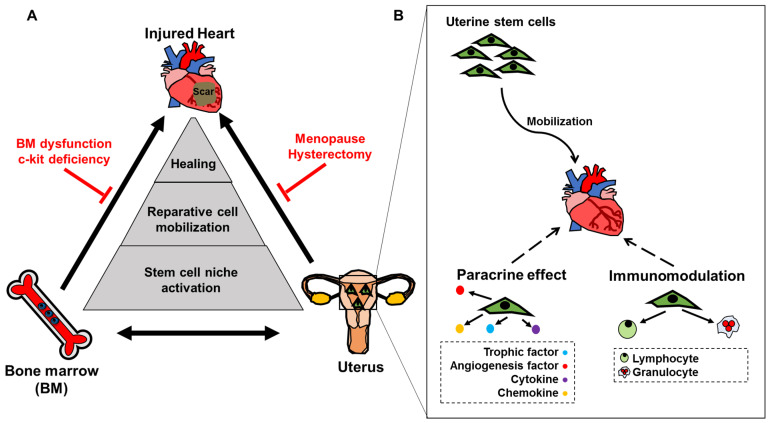
Relationship among the heart, uterus and bone marrow. (**A**) Schematic of the triangular relationship among heart, bone marrow, and uterus; the uterus is able to reciprocally interact with the BM to serve as a second stem cell reservoir. Cardiac injury triggers activation of both bone marrow and uterine stem cell niches, leading to mobilization of reparative cells from both niches, which traffic to the injury site to promote healing. (**B**) The utero-cardiac cross talk involves uterine stem cell mobilization to exert paracrine and immunomodulatory effects.

It is known that stem cell populations from the BM are mobilized in response to myocardial infarction and provide paracrine support to promote cardiac repair [71]. This BM-cardiac axis has been extensively studied [71,72,73,74,75]. Additionally, the existence of a BM-uterus axis has been described, and evidence exists for BM cell engraftment within the uterine tissue in response to injury [76]. All of these findings support the notion that stem cells may leave their niches in response to tissue damage to aid proper regenerative processes. We thus hypothesize that the uterus acts as an additional reservoir of stem cells, which is then able to migrate to distant bodily sites to promote healing. Evidence from our research group has demonstrated that uterine cells are capable of supporting hematopoiesis and reconstitute the BM of irradiated mice, establishing the utero-BM axis [69,70]. Our focus, therefore, is mainly to discuss the ability of uterine cells to contribute to cardiac repair post-myocardial infarction, via an utero-cardiac axis characterized by a three-way relationship among the heart, uterus, and BM (Figure 1). The other 2 axes (BM and heart) will only be briefly discussed, as they have already been discussed elsewhere.

### 4.1. Uterine Cells Traffic to the Injured Heart to Promote Healing: The Utero-Cardiac Axis

In response to an ischemic injury to the heart, the recruitment of regenerative cells has been demonstrated to be essential for recovery [71,77,78,79,80,81]. According to our research findings, females may have the advantage, owing to the proposed utero-cardiac axis, which provides additional support for cardiac healing [6,82,83,84]. Indeed, the uterus harbors a population of stem cells that can differentiate into myocyte, respiratory epithelial, neuronal, endothelial, hematopoietic, pancreatic, hepatic, adipocyte, and osteogenic lineages [85], as well as providing paracrine support for tissue repair [8,86]. In the context of myocardial infarction, our group has previously demonstrated that the uterus is the source of potent progenitor cells, which induce angiogenesis when injected into the infarcted heart [82,87]. Furthermore, we have shown that hysterectomized rats without oorphorectomy developed progressive cardiac dilatation and heart failure following myocardial infarction, whose severity was comparable to that of their male counterparts [6]. Transplanting a green fluorescent protein positive (GFP^+^) uterus, complete with vascular anastomosis, into a hysterectomized wild type recipient resulted in GFP^+^ cell recruitment into the heart, which rescued ventricular dysfunction after coronary occlusion, yielding ventricular functional parameters similar to those of non-hysterectomized females. In fact, GFP^+^ cells were found around blood vessels within recipient hearts, indicating that their support of angiogenesis is a primary effect of these cells [6]. Moreover, intravenous injection of uterine cells following hysterectomy and myocardial infarction enhanced tissue repair and prevented cardiac dysfunction, resulting in outcomes similar to that observed in non-hysterectomized animals. In an effort to explain the association between hysterectomy and adverse outcomes following myocardial infarction, we thus postulate that a functional uterus serves as a reservoir harboring regenerative cells. As mentioned above, stem cells are present within the uterus, and these cells have been tested for their effectiveness in various pathologies, including heart disease [88]. In fact, we have also previously demonstrated that uterine stem cells were able to support hematopoiesis in lethally-irradiated mice, in which some of the stem cells present were of extra-marrow origin, as demonstrated by our double-reconstitution study, in which the mice were first reconstituted with GFP^+^ BM cells, resulting in GFP being expressed in the majority of BM cells, but only for 6% of uterine cells within the recipients. Afterwards, the second reconstitution was carried out, involving a combination of uterine cells isolated from these chimeric mice, comprising a mixture of GFP^−^ native uterine cells and blood/BM-originating GFP^+^ cells. The recipient mice for the secondary reconstitution had no GFP^+^ cells in the BM, blood, or spleen, implying that the uterine cells obtained from the first reconstitution mice was likely not derived from BM cells [69,70]. This, along with the finding that GFP^+^ uterine stem cells were able to home into the heart following myocardial infarction to promote healing [6], among wild type animals that received a GFP^+^ uterine transplant, suggested that the uterus, like the BM, is able to export cells to the heart in response to injury. This relationship between the uterus and the heart, referred to as the “utero-cardiac axis”, could be disrupted by hysterectomy or uterine dysfunction (Figure 1).

The existence of this “utero-cardiac axis” is further supported by the observation that the homed uterine stem cells are closely associated with blood vessels. In fact, some uterine cells can be found integrating into vessel structures, and are detectable by endothelial cell markers [6]. Uterine cell trafficking into injured myocardium is also related to an increase in blood vessel densities [6], suggesting that these cells may have a pro-angiogenic function. Indeed, we successfully isolated a population of uterine stem cells from human endometrium, and found that they had greater proliferative, migratory and angiogenic capacities, with higher expression levels of pro-angiogenic factors, compared to human BM stem cells. Upon implantation into infarcted hearts, these pro-angiogenic cells maintained cardiac function, decreased infarct size and improved repair post-myocardial infarction, via increasing neo-vasculization and preserving viable myocytes [84]. This connection between uterine stem cells and increased angiogenesis is not surprising, as the uterus is a rare and abundant site of non-pathological angiogenesis in the post-development adult body. This is especially true in menstruating species, including humans, who undergo repeated cycles of endometrial-decidual angiogenesis. Therefore, stem cells or reparative cells with pro-angiogenic function may represent a unique population of uterine cells that are able to be exported in response to cardiac injury, which may serve as an additional axis of cell mobilization present in women to complement BM support.

A summary of current literature on the uterus as a stem cell reservoir and its cardio-protective effects is listed in Table 1.

### 4.2. The BM-Cardiac Axis

The BM-cardiac axis has been extensively studied and reviewed elsewhere in the literature [97]. BM serves as a reservoir of stem and progenitor cells, which differentiate to produce circulating blood and immune cells. During this process, stem cells from the BM cross talk with peripheral organs and influence their functioning. With respect to myocardial infarction, we observed in our previous studies that the BM exports stem cells to the site of injury, which then potentiate the cardiac repair process by inducing angiogenesis, preventing matrix remodeling, and activating endogenous precursors. On the other hand, dysfunctional BM stem cells, with c-kit receptor mutants, hindered recovery following myocardial infarction, which was reversed via reconstitution of the BM with wild-type BM stem cells [71,72]. All these findings demonstrate the presence of a BM-cardiac axis, as well as highlighting the importance of a functional BM for supporting cardiac repair. This axis is particularly important among males, as it serves as the only major route for promoting cardiac regeneration, as the heart itself only has a limited self-reparative capacity.

In light of these aforementioned findings, various therapies have been developed, in which BM cells are directly injected into the myocardium after injury, in hopes of yielding the same outcomes as that of natural mobilization, where BM cells end up in the injured area and promote cardiac repair. Intra-myocardial transplantation using autologous CD133 BM cells have been shown to be safe and feasible for patients undergoing coronary artery bypass grafting [98]. Another approach, aside from direct myocardial injections, is to replace dysfunctional stem cells, such as aged ones, with functional ones, such as those obtained from younger donors, through BM reconstitution. This reconstitution with young BM stem cells resulted in the restoration of cardiac reparative capabilities post-myocardial infarction, owing to those cells expressing factors promoting the activation of repair-oriented signaling pathways [73,74,75]. Currently, several clinical trials are ongoing, utilizing different approaches with BM-derived stem cells for cardiac repair to determine their effectiveness.

### 4.3. Utero-BM Axis

It is well accepted that BM stem cells can differentiate into both hematopoietic and mesenchymal lineage cells, suggesting that these cells may not only contribute to cardiac regeneration after injury, but also to the maintenance of multiple other tissues, such as in the uterus. In fact, although uterine stem cells are likely derived from residual fetal stem cells [99], evidence exists indicating that BM-derived cells can contribute to endometrium regeneration [41,76]. Taylor et al. reported the presence of donor-derived endometrial stromal and epithelial cells within the biopsied tissues of four women who received single-HLA antigen mismatched BM transplants [41]. Further studies performed in mice using gender-mismatched BM transplants have shown evidence of stromal and epithelial cells containing the Y chromosome, derived from male BM stem cell donors, within the uterine tissue of female mice [76]. In fact, the involvement of BM cells within the uterus has been found to be instrumental for both physiological and pathological processes. For instance, the chemokine C-X-C motif chemokine ligand 12 (CXCL12) has been found to play a major role in recruiting BM-derived progenitor cells into the uterus during pregnancy in mice, via its interaction with the C-X-C chemokine receptor type 4 (CXCR4) receptor on these cells. This recruitment, in turn, facilitates the formation of the characteristic decidual structures of the pregnant endometrium, which is essential for ensuring successful implantation of the fetus in the uterus [100]. Even under non-pregnant conditions, these BM-derived progenitor cells are still able to traffic into the uterus and differentiate into cells similar to that of endogenous uterine cells, such as the endometrium, in a BM replacement study [101]. Indeed, BM-derived progenitor cells are involved in the post-partum remodeling of the uterus [102,103], as well as being able to repair damaged endometrium in a rat model [104]. With respect to pathological processes, one of the most popular theories for the etiology of endometriosis, the formation of endothelial tissue outside of its appropriate location in the uterus, has been owed to the trafficking of stem cells from the BM to other bodily sites, followed by differentiation of these cells into endometrial cells, at locations outside the uterus [105]. However, whether these findings are also applicable to humans, especially when accounting for the differences between mouse estrous and human menstruation periods, should be further investigated.

Additionally, this cross talk between the BM and the uterus could possibly go both ways, as our study demonstrated that the uterus contains a population of hemangioblasts [69], with an extra-medullary origin. These HSC-like hemangioblasts were able to reconstitute the BM of lethally-irradiated mice [70]. We also excluded the possibility that these results are due to BM cells residing in the uterus. Thus, the uterus can be considered as another reservoir for stem cells, and could reciprocally interact with the BM.

## 5. The Implications of the Utero-Cardiac Axis with Respect to Preventive Hysterectomy

Women with a high predisposition for cancer, associated with breast cancer type 1/2 susceptibility protein (*BRCA1* and *BRCA2*) mutations, have the option of surgically remove their uterus, breasts or ovaries as a preventive strategy. The hypothesized impact on preventive surgery was studied by Borzekowski et al., who found that 57% of women surveyed in the United States would choose preventative surgery if they were found to be genetically at risk [106]. However, this reduction of cancer risk by prophylactic surgery was noted by Centerwall et al. to be outweighed by increased risk of death from cardiovascular diseases [62]. In light of the existence of the utero-cardiac axis, caution should be taken for preventative hysterectomy, as it may eliminate a niche of reparative cells that can contribute to improved cardiac function and survival in the aftermath of heart diseases.

## 6. The Effects of the Utero-Cardiac Axis for Developing Uterine Cell Therapies

Currently, BM mesenchymal stem cells are the preferred approach for developing therapies for treating cardiac disease [107,108], owing to their ability to home to the heart and promote reparative processes [71,72,109]. Based on this logic, uterine stem cells could also be utilized in analogous ways as that of BM mesenchymal stem cells, as they are also able to migrate to the heart in response to injury. Indeed, the similarities in their migratory and cardiac repair capabilities have led to the possibility of intra-myocardial transplantation of uterine stem cells being a potential treatment option for heart disease, lowering the need for invasive surgical procedures [82,84,86,110,111].

Both uterine and BM stem cells, being categorized as mesenchymal stem cells, share great similarities, but also significant differences, from several aspects: (1) Phenotypically, they both express mesenchymal stem cell linage markers (e.g., CD29, CD44, CD73, CD90, and CD105) but are devoid of hematopoietic linage and immuno-markers (CD14, CD34, CD45, and HLA-DR) [45]. (2) They both have the ability to differentiate into the three classical lineages: osteocytes, chondrocytes and adipocytes. However, a previous study we conducted has shown that compared to BM mesenchymal stem cells, the adipogenic differentiation ability is significantly lower in uterine mesenchymal stem cells, while similar osteogenic differentiation ability is present between both cells [84]. (3) Both cells express similar secretory factors and possess immuno-privilege properties. However, compared to BM mesenchymal stem cells, uterine mesenchymal stem cells secrete lower levels of multiple cytokines, such as VEGF-A, SDF-1α, IL-1RA, IL-6, interferon gamma-induced protein 10 (IP-10), monocyte chemoattractant protein-1 (MCP-1), macrophage inflammatory protein-1α (MIP-1α), and regulated on activation, normal T cell expressed and secreted (RANTES) [112], as well as higher levels of IL-8, along with up to 100,000-fold higher amounts of pro-reparative factors, such as matrix metalloproteinases 3 and 10 (MMP-3, -10), granulocyte macrophage colony-stimulating factor (GM-CSF), angiopoietin-2 and platelet-derived growth factor BB (PDGF-BB) [108]. We believe that these subtle, yet substantial secretory differences between uterine and BM stem cells may contribute to the greater paracrine support capabilities of the former, particularly with respect to angiogenesis. This greater ability for supporting angiogenesis is further facilitated by uterine stem cells expressing significantly higher levels of Fms-like tyrosine kinase 1 and FGF9 [84]. (4) With respect to the feasibility for clinical application, compared to BM stem cells, it is easier to harvest uterine stem cells for cell therapy strategies, especially in the case of menstrual blood-derived endometrial stem cells, owing to their abundant source material and capability to easily obtain regular donations, along with their superior proliferative and migratory capacities, as well as their ability to be used for autologous transplantation. All of these characteristics are advantageous over BM stem cells.

The pro-reparative effects of uterine stem cells are widely attributed to paracrine mechanisms [113,114]. In fact, the paracrine properties of such cells has resulted in them being widely investigated in clinical trials for treating heart failure [88]. Furthermore, endometrial stromal stem cells from pigs were found to be able to differentiate into cardiomyocyte-like cells in vitro, possibly providing a second route for applying uterine stem cells in a therapeutic context [93]. As a result, these cells could serve as producers of paracrine factors to support engineered-tissue grafts and the differentiation of other pluripotent stem cells into cardiac tissue for reparative procedures, as well as being a pluripotent cell source itself. However, these cardiomyocyte-like cells were still far from being mature cardiomyocytes with fully functional electrophysiological and contractile characteristics [93]. Therefore, the participation of uterine stem cells in the cardiac repair process is likely more through immunomodulatory and angiogenic effects promoting the survival of the remaining cardiomyocytes within the recipient, rather than direct differentiation of those cells into cardiomyocytes.

Other studies have found that uterine stem cells could be utilized for treating other diseases, suggesting that they are versatile in function. In fact, preliminary data have already outlined that they are able to restore dopamine function in a mouse model of Parkinson’s disease [115], as well as treating diabetes [116] and critical limb ischemia [117]. Additional studies have shown that uterine stem cells were able to support cardiac allografts via secreting paracrine factors, such as stromal cell-derived factor 1 (SDF-1) and galectin-1 [79,82]. All these findings, therefore, suggest that uterine stem cells are effective as cell therapeutic agents.

It is worth noting, though, that like with BM stem cells, a number of stressors could negatively impact the therapeutic efficacy of uterine stem cells, such as aging or pathological damage to the uterus. With respect to aging, it has been noted to play a significant role regarding the decline in endometrial stem cell functioning over time. The manifestation of an aging phenotype has been linked to oxidative stress, which leads to the up-regulation of genes favoring, and down-regulation of genes acting against, senescence-associated processes. One example is with sonic hedgehog signaling, which is found to be lowered among aged uterine stem cells, thereby releasing its suppression on the aging-promoting factor serpin family B Member 2 (SERPINB2). In fact, exogenous application of sonic hedgehog on aging uterine stem cells reverses their age-associated functional declines. Another oxidative stress-induced gene is insulin like growth factor binding protein 3 (*IGFBP3*), which promotes premature senescence in uterine stem cells, either in an IGF-1-dependent or independent manner; inhibition of IGFBP3 reversed uterine stem cell senescence [118,119]. As for pathological damage, it has been noted that damage to the deep layers of the endometrium could potentially affect the resident stem cells, resulting in decreased cell numbers and capabilities, thus leading to the failure of functionalis regeneration. This lack of regeneration subsequently serves as a contributing factor behind the onset of intrauterine adhesion, or Asherman syndrome, characterized by the replacement of endometrial tissue with fibrous tissue [120]. Therefore, these factors possibly affecting the efficacy of uterine stem cells need to be taken into account before their application for clinical cell therapies.

Another interesting observation is the possible parallel role of polyploidy among both cardiomyocytes and uterine stem cells, which is associated with the cession of stem cell regeneration. In cardiomyocytes, cell numbers only increase during fetal development; afterwards, cardiac expansion switches from hyperplasia to hypertrophy, entailing increases in cell sizes [121,122]. Hypertrophy could occur under both physiological, such as exercise [123], as well as pathological, such as myocardial infarction, conditions [124]. Owing to the association of polyploidy with hypertrophy, it has often been considered as a significant barrier against cardiomyocyte proliferation and subsequent heart regeneration [125]. Polyploidy is also found among uterine decidual cells, which plays a significant role in preparing for blastocyst implantation [126]. Studies have shown that stromal cells first differentiate into decidual cells to form the primary decidual zone [127,128,129], followed by poly-ploidization of adjacent decidual cells to form the secondary decidual zone [128]. These cells, similar to cardiomyocytes, are terminally-differentiated, and later undergo apoptosis, replaced by non-polyploid decidual cells in both decidual zones before placenta formation [130]. Therefore, polyploidy among decidual cells, similar to cardiomyocytes, could serve as a limiting factor against continued decidual cell growth to facilitate embryonic implantation. Overall, polyploidy among both cardiomyocyte and uterine stromal cells share the characteristic of being the hallmark of the cession of cell proliferation and regeneration. However, cardiomyocyte polyploidy is more associated with pathophysiological cardiac hypertrophy, while among uterine stem cells, polyploidy is seen as more a part of the physiological process for embryonic implantation.

The paracrine-based therapeutic benefits of uterine stem cells may possibly be achieved by cell-derived extracellular vesicles. In fact, multiple studies have shown that these vesicles possess pro-angiogenic, anti-apoptotic, and immunomodulatory effects. A study using high-throughput proteomic analysis reveals that ~617 proteins from endometrial stem cells were annotated as originating from extracellular exosomes, comprising ~70% of the total extracellular vesicular proteome [131]. The vesicles are also enriched with miRNAs, whose preferred target site, according to next-generation sequencing, is the nucleus, through which they are able to regulate signal transduction, cell proliferation, and apoptotic processes [131]. These results allow us to understand the complexity of the molecular networks in uterine stem cell-derived extracellular vesicles and their potential effects on target cells. The application of these vesicles and their contents has also been observed in other studies, such as one where menstrual stem cell-derived small extracellular vesicles are able to alleviate intrauterine adhesion-associated uterine damage, recovering proper endometrial morphology, reversing fibrosis, and promoting gland regeneration and angiogenesis [132], probably through inhibiting TGFβ1/SMAD3 signaling, along with promoting SMAD1/5/8 and ERK1/2 phosphorylation, as well as upregulating BMP7 [132]. In fact, these vesicles are more effective in treating intrauterine adhesion compared to cell therapies, as their effects are more localized within the endometrium [132]. Extracellular vesicles may also exert their effects through miRNAs, such as exosomal Let-7, which is able to decrease pulmonary fibrosis via alleviating mitochondrial DNA damage [133], as well as miR-21, which serves as the basis behind the greater cardioprotective capabilities and angiogenesis increases associated with endometrial stem cells, through its activation of PTEN/Akt pathway [90]. Collectively, the therapeutic values of uterine stem cell-derived extracellular vesicles remain a relatively under-explored venue; future studies are thus warranted to fully elaborate on whether these vesicles could serve as a valuable alternative for cell therapies, owing to lower expenses and ease in application.

In summary, uterine stem cells are a unique type of stem cells, which have been demonstrated to be an effective potential therapeutic approach for treating various diseases, including cardiac disease. However, further work is necessary to determine their true effectiveness, particularly with respect to the cardiovascular system, and the mechanisms that could really unlock the potential of the utero-cardiac axis for alleviating heart disease.

## 7. Conclusions

As we deepen our understanding of the fluidity and plasticity of the human body, the existence of an exchange of stem cells between organs to either maintain the pool of stem cells within these organs under physiological conditions, or support proper regeneration after injury, may provide a new therapeutic approach for treating various pathological conditions. Here, we show additional evidence that not only BM, but also the uterus can be considered a stem cell reservoir, which is able to actively export cells to distant targets, such as the heart. This migration of stem cells to the heart, in turn, is able to promote angiogenesis and formation of repair tissue, leading to improvements of cardiac functioning. However, future research should investigate the exact mechanisms behind this utero-cardiac axis, and how it could be manipulated to potentiate the beneficial effects for treating heart diseases.

## Figures and Tables

**Table 1:** Utero-cardiac cross talk: Current literature on the uterus as a stem cell reservoir and its cardio-protective effects.

	Method	Result	Reference
Rats who undergone hysterectomy, followed by heterotropic GFP^+^ uterine transplant and MI	GFP^+^ uteruses were removed from GFP rats and heterotropically-transplanted into non-GFP recipients who have undergone hysterectomy; MI was then induced	Heterotropic-transplanted uterine GFP^+^ cells were found in recipient hearts 7 days after MI and persisted for 6 months, in which they were localized around blood vessels, and improved cardiac functioning	[6]
Commercially-obtained menstrual blood-derived mesenchymal stem cells and a rat MI model	2 × 10^6^ menstrual blood-derived mesenchymal stem cells were intramyocardially-injected into a Sprague-Dawley rat MI model	Menstrual blood-derived mesenchymal stem cells improved cardiac functioning through inhibition of the TGF-β/Smad-induced endothelial to mesenchymal transition, in turn reducing cardiac fibrosis	[89]
Murine uterine MHC I^−^ and MHC I^+^ cells, along with a murine MI model	Murine uterine MHC I^−^ and MHC I^+^ cells were isolated from C57BL/6N mice, characterized in vitro for their immuno-modulatory properties, followed by allogenic injection of 0.5 × 10^6^ cells into a FVB mouse MI model	MHC I^−^ cells were immuno-privileged, with lower cell death and leukocyte proliferation, as well as yielding comparable benefits to syngeneic bone marrow cell transplantation after intra-myocardial injection, with engraftment in cardiac tissue and limited recruitment of CD4 and CD8 cells	[82]
Rat uterine-derived CD11b cells and a rat ischemia/reperfusion model	9 × 10^5^ CD11b^+^ cells were intramyocardially injected into ischemic/re-perfused rat hearts 5 days post-injury	CD11b cells increased vasculogenesis, leading to reduced infarct size, as well as restoring myocardial functioning and perfusion	[83]
Human endometrium-derived, bone marrow, and adipose-derived mesenchymal stem cells in a rat MI model	Human endometrium-derived, bone marrow, and adipose-derived mesenchymal stem cells were injected intra-myocardially to compare their cardio-protective capabilities	Endometrium-derived mesenchymal stem cells had greater cardioprotective capabilities and increased angiogenesis via secreting miR-21 in exosomes, which in turn activates the PTEN/Akt pathway	[90]
Murine heart transplant model and human menstrual blood-derived ERC	Heterotropic cardiac transplantation was conducted from C57BL/6 to BALB/c mice, followed by intravenous injection of 1 × 10^6^ human ERCs	ERC treatment prolonged cardiac allograft survival in mice by reducing CD19^+^ B cell numbers and activity	[91]
Murine heart transplant model and human ERCs	Heterotropic cardiac transplantation was conducted from BALB/c to C57BL/6 mice, followed by intravenous injection of 1 × 10^6^ human ERCs	Inhibition of ERC-produced SDF-1by the antagonist AMD3100, resulted in cardiac allograft rejection in recipient mice, as it was associated with increased antibodies and infiltrating immune cells	[92]
Porcine adipose-derived and endometrial stromal mesenchymal stem cells	Endometrial stromal and adipose-derived mesenchymal stem cells were obtained from pigs, and their markers, growth, and differentiation potential were compared to each other in vitro.	Endometrial stromal mesenchymal stem cells had higher growth rates compared to adipose-derived mesenchymal stem cells, as well as being able to differentiate into cardiomyocyte-like like cells	[93]
Human decidual stem cells from the first trimester of pregnancy and bone marrow stem cells in a rat MI model	Human CD34^+^ decidual stem cells were obtained from women who terminated during the first trimester, and compared to bone marrow stem cells obtained from cardiac surgery patients	Human CD34^+^ decidual stem cells had greater angiogenic capabilities, compared to bone marrow stem cells, as well as being able to increase cardiomyocyte survival and increase neo-vasculature post-MI	[94]
Murine heart transplant model and human ERCs	Heterotropic cardiac transplantation was conducted from BALB/c to C57BL/6 mice, followed by intravenous injection of 5 × 10^6^ human ERCs	Human ERCs expressed Galectin-9, which suppressed immune responses, in the form of lowered Th1, Th17, CD8^+^ T, and B cell activity, decreased donor-specific antibody levels, and enhanced Treg, all of which contributed to prolonged cardiac allograft survival	[95]
Murine heart transplant model and human ERCs	Cardiac allograft transplantation was conducted from BALB/c donors to C57BL/6 mice, followed by implantation of human endometrial stem cells, either untreated or pre-treated with CD73 monoclonal antibodies	CD73 on ERCs led to decreased pro-inflammatory cytokines IFN-γ and TNF-α, increased anti-inflammatory cytokine IL-10, as well as increasing expression of protective cardiac allograft receptor A_2B_. By contrast, blocking CD73 led to reduced Tol-DC, M2, and Treg activity	[96]
Human endometrial and bone marrow stem cells in a rat MI model	Human endometrial stem cells were isolated from 22 premenopausal women, and compared to human bone marrow mesenchymal stem cells derived from 25 age-matched patients	Human endometrial stem cells had greater proliferative, migratory, and pro-angiogenic capabilities, as well as being able to preserve viable cardiomyocytes and improve cardiac functioning post-ischemic injury, compared to bone marrow stem cells	[84]

ERC: endometrial regenerative cell; IFN: interferon; MHC: major histocompatibility complex; MI: myocardial infarction; PTEN: phosphatase and tensin homolog; Th: T helper type cell; TNF: tumor necrosis factor; Tol-DC: tolerogenic dendritic cell; Treg: regulatory T cell.
